# Leukotoxin A Production and Release by JP2 and Non-JP2 Genotype *Aggregatibacter actinomycetemcomitans* in Relation to Culture Conditions

**DOI:** 10.3390/pathogens13070569

**Published:** 2024-07-06

**Authors:** Sotirios Kalfas, Zahra Khayyat Pour, Rolf Claesson, Anders Johansson

**Affiliations:** 1 Department of Preventive Dentistry, Periodontology and Implant Biology, Aristotle University of Thessaloniki, 54124 Thessaloniki, Greece; kalfas@dent.auth.gr; 2 Department of Odontology, Umeå University, 90187 Umeå, Sweden; z.khayyatpour@allt1.se (Z.K.P.); rlkc1952@gmail.com (R.C.)

**Keywords:** *Aggregatibacter actinomycetemcomitans*, leukotoxin release, JP2-genotype, culture conditions

## Abstract

Aggressive forms of periodontitis, especially in young patients, are often associated with an increased proportion of the Gram-negative bacterium *Aggregatibacter actinomycetemcomitans* of the microbiota of the affected periodontal sites. One of the virulence factors of *A. actinomycetemcomitans* is a leukotoxin (LtxA) that induces a pro-inflammatory cell death process in leukocytes. *A. actinomycetemcomitans* exhibits a large genetic diversity and different genotypes vary in LtxA production capacity. The genotype JP2 is a heavy LtxA producer due to a 530-base pair deletion in the promoter for the toxin genes, and this trait has been associated with an increased pathogenic potential. The present study focused on the production and release of LtxA by different *A. actinomycetemcomitans* genotypes and serotypes under various growth conditions. Four different strains of this bacterium were cultured in two different culture broths, and the amount of LtxA bound to the bacterial surface or released into the broths was determined. The cultures were examined during the logarithmic and the early stationary phases of growth. The JP2 genotype exhibited the highest LtxA production among the strains tested, and production was not affected by the growth phase. The opposite was observed with the other strains. The composition of the culture broth had no effect on the growth pattern of the tested strains. However, the abundant release of LtxA from the bacterial surface into the culture broth was found in the presence of horse serum. Besides confirming the enhanced leucotoxicity of the JP2 genotype, the study provides new data on LtxA production in the logarithmic and stationary phases of growth and the effect of media composition on the release of the toxin from the bacterial membrane.

## 1. Introduction

The Gram-negative facultative anaerobic bacterial species *Aggregatibacter actinomycetemcomitans* is associated with periodontitis in young individuals [1,2]. A leukotoxin (LtxA) produced by this bacterium is closely linked to the initiation of degenerative processes involved in the disease [3,4]. LtxA is a large pore-forming protein internalized by the β_2_ integrin LFA-1 (CD11a/CD18) expressed by human immune cells [5]. The interaction of LtxA with leukocytes activates the release of interleukin (IL)-1β from human macrophages and of proteolytic enzymes from neutrophils [6] and ultimately causes cell death. LtxA-induced macrophage death involves the activation of the NLRP3 inflammasome in a process defined as pyroptosis [7]. The NLRP3 inflammasome plays a central role in many degenerative diseases and is a possible target for future strategies in periodontal therapy [8]. *A. actinomycetemcomitans* shows a substantial genetic diversity and seven serotypes (a–g) have been described till now [9,10]. A specific variant of serotype b, the JP2 genotype, produces large amounts of LtxA and is characterized by the absence of a 530-base pair (bp) sequence within the *ltxCABD* promoter region [11]. 

The JP2 genotype was initially detected in individuals with origins in the Mediterranean part of Africa, following a dissemination route through West Africa, and further to North and South America via the transatlantic slave trade [12]. Today, there are several reports on the carriage of the JP2 genotype by individuals outside the North and West African regions [13]. Young carriers of the JP2 genotype of *A. actinomycetemcomitans* are at high risk of developing periodontitis [14]. In addition to the JP2 genotype, an additional marker for highly leukotoxic genotypes of *A. actinomycetemcomitans* has been discovered [15]. This genotype contains an intact *cagE* gene and includes JP2, as well as a subgroup of non-JP2 isolates with genetic similarities to JP2 [16].

LtxA production substantially varies among different genotypes, being highest in strains of the JP2 genotype [17]. The role of the 530 bp deletion in the promoter region of the leukotoxin operon for the regulation of LtxA expression is not fully understood [18]. The deletion of a specific 100 bp region within the 530 bp promoter deletion region was shown to be a leukotoxin repressor, thus enhancing LtxA production [19]. As a supplement to this finding another recently discovered leukotoxin promoter deletion outside this 100 bp deletion also resulted in enhanced LtxA production in the JP2 genotype, further illustrating the complexity of leukotoxin production [20]. These discrepancies in the role of the promoter deletion for LtxA production indicate a complex regulation with alternative signaling pathways. Environmental factors and growth conditions have also been described as interfering with LtxA production [21,22,23].

LtxA is a 1055 amino acid protein post-translationally activated through acylation before being secreted by a Type I secretion system [24]. The secreted LtxA may either be released into the culture media or stay bound to the bacterial outer membrane [25,26]. The presence of serum proteins induces the release of LtxA from the bacterial outer membrane [27]. The ionic strength of the environment also affects this release [28]. Serum protease inhibitors protect LtxA from proteolytic degradation, which, in turn, promotes increased leukotoxic activity [29].

The aim of the present study was to examine LtxA production by the JP2 and non-JP2 genotypes of *A. actinomycetemcomitans* in relation to certain culture conditions, such as the culture medium and the phase of growth.

## 2. Materials and Methods

### 2.1. Culture Media and Bacterial Strains 

Two sterile-filtered culture broths, peptone yeast extract glucose (PYG) [4] and Bacteroides medium (BM) [30] were used. Their composition is shown in Table 1. All chemicals were obtained from Sigma-Aldrich (St. Louis, MA, USA). 

The growth of four *A. actinomycetemcomitans* strains, NCTC 9710, HK 1519 (JP2 genotype), SUNY ab75, and Y4, isolated from human plaque, was initially examined in the two broths (Table 2). A total of 1 mL of an overnight culture was inoculated into 50 mL of fresh broth and incubated at 37 °C in air with 5% CO_2_. The bacterial growth was followed by measuring the optical density at 600 nm (OD_600_) of the culture at certain time points. The growth was followed for 24 h.

For the analysis of LtxA production, half of the culture was harvested in the late logarithmic phase of growth after about 8 h of incubation and the rest of the culture after an additional 12 h incubation, i.e., in the stationary phase of growth. 

### 2.2. Extraction of LtxA from Bacterial Cells

Each culture sample was immediately centrifuged at 10,000× *g* and 4 °C for 30 min, and the bacterial pellet was re-suspended in 50 mM phosphate buffer containing 0.45 M NaCl, pH 7.0, and the density was adjusted to 3 × 10^11^ cells/mL. The suspension was incubated under gentle rocking at 4 °C for 1 h to release LtxA and other outer membrane proteins from the bacterial cells [28]. At the end of the incubation, the cells were removed by centrifugation (10,000× *g* at 4 °C for 20 min), and the supernatant was stored at −80 °C until analyzed.

### 2.3. Precipitation of LtxA Released into the Culture Medium

LtxA and other proteins released from the bacteria into the broth were precipitated by the addition of trichloroacetic acid to the culture supernatant to a final concentration of 5% and incubated overnight at 4 °C. The precipitate was harvested by centrifugation (3000× *g* at 4 °C for 15 min) and resuspended in 0.1 M NaOH to a final volume that corresponded to protein extraction from 3 × 10^11^ bacteria/mL, this concentration being comparable with the one in LtxA extracts from the bacterial cells. The solution was stored at −80 °C until analyzed.

### 2.4. Detection of LtxA in Electrophoresis Gel

The proteins in the precipitates of culture broths and in cell extracts were separated by sodium dodecyl sulphate polyacrylamide gel electrophoresis (SDS-PAGE, 8%). Each sample was mixed with an equal volume of buffer (50 µL 50 mM Tris-buffer, pH 7.4, containing 2% SDS, 0.03% bromophenol blue, 20% sucrose, and 5% mercaptothion) and heated at 100 °C for 5 min. The protein bands in the gel were stained with Coomassie brilliant blue R-250 (Bio-Rad Laboratories, Hercules, CA, USA) and the amount of protein in the 116 kDa band that corresponds to leukotoxin was coarsely determined with the Model Gs-700 Imaging Densitometer and the Molecular Analyst™ software version 1.4 (Bio-Rad Laboratories).

### 2.5. Detection of LtxA by Western Blot Analysis and Amino Acid Sequencing

The proteins in the SDS-PAGE were transferred from the gel to a Polyscreen™ transfer membrane (NEN Life Science Products, Mechelen, Belgium) as previously described [28]. The membrane was incubated in a TBS buffer (20 mM Tris and 500 mM NaCl, pH 7.5) containing rabbit anti-leukotoxin serum (diluted 1:1000) for 1–2 h. As a secondary antibody, goat anti-rabbit HRP (DAKO A/S. Glostrup, Denmark) was used (diluted 1:1000 in TBS-buffer) for 1 h. The immunoreactive bands were visualized on film (Kodak) with ECL development solution according to the manufacturer’s instructions (PIERCE, Rockford, IL, USA).

The N-terminal amino acid sequence analysis of the 116 kDa protein band, transferred from the gel to a Polyscreen™ membrane, was performed with the Procise™ 494 (Applied Biosystems, Foster City, CA, USA) protein sequencing system, using programs and chemicals recommended by the manufacturer [28].

### 2.6. Isolation of PMN from Peripheral Blood

Human polymorphonuclear leukocytes (PMNs) were prepared from peripheral blood collected in sodium heparin-containing vacuum tubes from two healthy donors (co-authors RC and AJ). After sedimentation in Macrodex (Pharmacia, Uppsala, Sweden), the upper phase was centrifuged (150× *g* at 4 °C for 12 min) and the cell pellet was resuspended in distilled water for 30 s to lyse remaining red blood cells. Thereafter, NaCl was added to make the solution isotonic, and the cells were washed twice with PBS before being resuspended in RPMI medium with 20% fetal calf serum. The cell concentration in the suspension was determined in a Bürker chamber under 400× magnification and adjusted to 4 × 10^6^ cells/mL. The final preparation contained 90–95% PMN cells. 

### 2.7. Assay of Leukotoxic Activity

The leukotoxicity of the various samples was determined by the activity of lactate dehydrogenase [28] released from injured PMN cells upon exposure to the samples. A volume of 75 µL of PMN suspensions (2 × 10^6^ cells/mL) was mixed with an equal volume of bacterial cell suspensions in RPMI with 20% FCS. Mixtures with a bacterial/PMN ratio of 3, 6, 12, 25, 50, 100, and 200 were prepared and incubated at 37 °C in air with 5% CO_2_ for 2 h. The leukocytes were pelleted by centrifugation (250× *g* for 5 min), and the activity of lactate dehydrogenase released in the supernatant was determined [31]. The positive and negative controls were PMN lysed with 0.1% Triton X-100 and PMN only in RPMI, respectively.

A leukotoxicity assay was run in triplicate in a 96-well flat-bottomed microtiter plate. The reaction mixture contained 20 µL sample (supernatant from the centrifuged mixtures) and 180 µL LDH-buffer (0.1 M sodium phosphate buffer pH 7.0, with 2 mM Na-pyruvate and 0.154 mM NADH-Na). The activity of LDH was monitored at room temperature in a spectrophotometer (Multiscan MCC/340, Labsystems Diagnostic Oy, Vantaa, Finland) by continuously measuring the change in absorbance at 340 nm, indicative of NADH oxidation, for 2–5 min. The LDH activity of each sample was recorded as the decrease in absorbance/minute (DA) and the relative leukotoxicity (RL) of each sample was expressed in percent (%) and calculated by the formula:

RL (%) = 100 × (DA_sample_ − DA_neg.control_)/(DA_pos.control_ − DA_neg.control_) (1)

## 3. Results

### 3.1. Bacterial Growth

**Figure 1:**
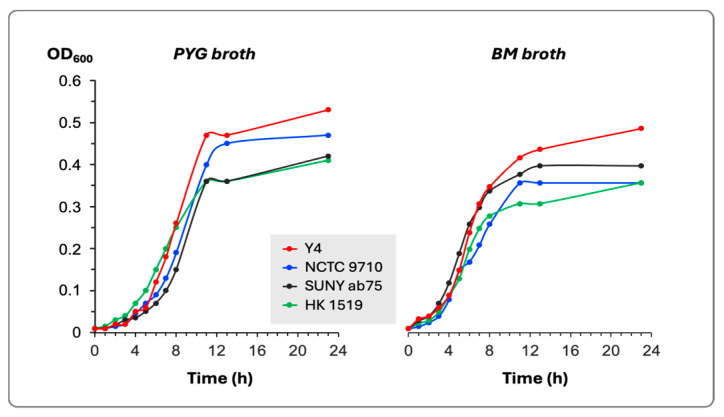
Growth curves in PYG and BM broths of the four bacterial strains. The growth was determined by the change in the optical density of the culture at 600 nm (OD_600_).

The estimation of bacterial growth in two different culture media did not show any substantial differences among the four tested bacterial strains (Figure 1). All strains reached the stationary phase of growth after 9–11 h of incubation at 37 °C.

The generation time for each bacterial strain in both culture media was calculated and presented in Table 2.

### 3.2. Cell Surface-Associated LtxA

The extraction of leukotoxin from bacterial cells showed that strains HK 1519 and Y4 produced high amounts of LtxA during the log phase. The LtxA production by the NCTC 9710 and SUNY ab75 strains was very low or undetectable. During the stationary phase, the HK 1519 strain exhibited more LtxA per bacterial cell than during the log phase, while Y4 showed no change in its content of leukotoxin. 

**Figure 2:**
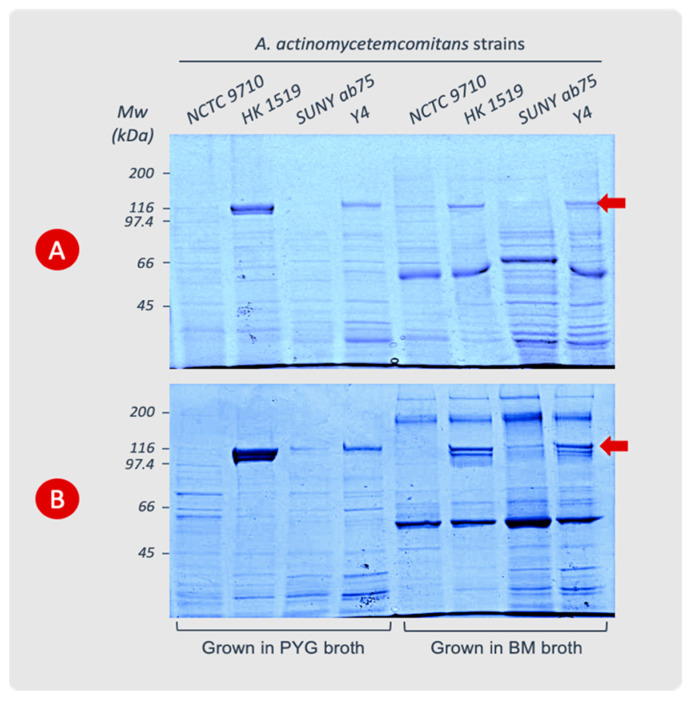
Cell surface-associated LtxA extracted from *A. actinomycetemcomitans* strains cultured in PYG or BM broth. The cells were harvested during the logarithmic (**A**) or stationary (**B**) phases of growth. The leukotoxin appears as a protein band at the 116 kDa position (arrow) in SDS-PAGE (8%). Data from representative experiments are shown.

In the log phase, both HK 1519 and Y4 had more leukotoxin associated with the bacterial cells in PYG than in BM. On the other hand, during the stationary phase, strain HK 1519 released more leukotoxin in PYG than in BM, while Y4 had more leukotoxin in BM media compared with PYG (Figure 2 and Table 3). Within the quantitative limitations of the method, the JP2 genotype (strain HK 1519) appeared to continue producing high amounts of LtxA also in the stationary phase of growth, while a more variable LtxA production was observed in the other non-JP2 strains (Table 3). This phenomenon seemed most pronounced in PYG broth.

An abundance of LtxA compared with the other proteins in the cell extracts from the JP2 genotype was observed (Figure 2). The analysis of leukotoxin precipitated from the supernatants of the different bacterial cultures showed very low or no detectable amount of leukotoxin in SDS-PAGE gels. 

**Figure 3:**
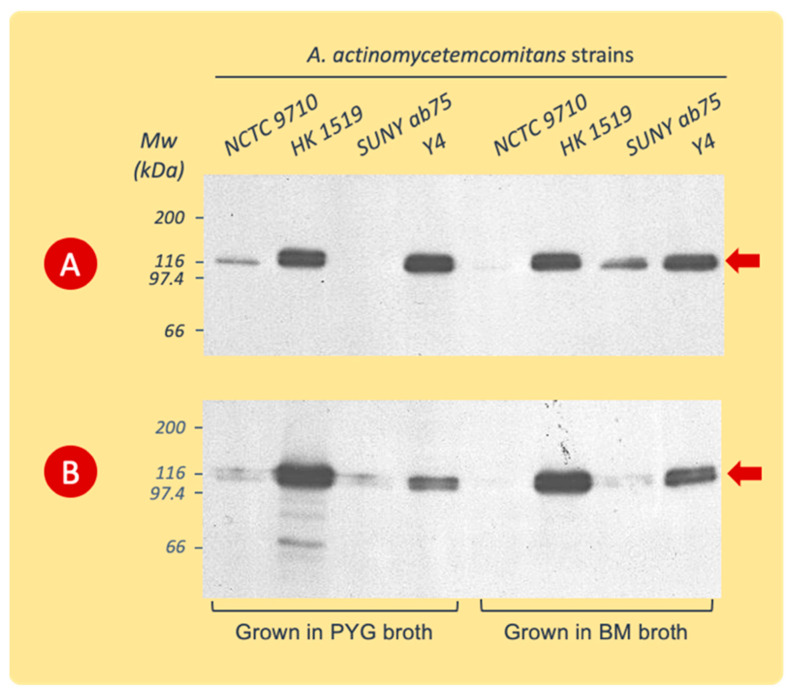
Western blot analysis (with polyclonal rabbit LtxA antiserum) of cell surface-associated LtxA extracted from *A. actinomycetemcomitans* strains cultured in PYG or BM broth. The cells were harvested during the logarithmic (**A**) or stationary (**B**) phases of growth. Data from representative experiments are shown. The leukotoxin appears at the 116 kDa position (arrow).

The immunoblotting of gels with the SDS-PAGE separated extracts showed a high specificity of the polyclonal leukotoxin antiserum for the 116 kDa protein band (Figure 3). The amino acid sequence analyses of this protein band showed a complete sequence homology with *A. actinomycetemcomitans* LtxA, (accession number P16462, https://www.uniprot.org/uniprotkb/P16462/entry, accessed on 7 January 2015).

### 3.3. LtxA Released in the Culture Broth

The detection of LtxA released into the growth broth was accomplished by the immunoblotting of the proteins precipitated from the culture supernatants and separated by SDS-PAGE, as described above. 

**Figure 4:**
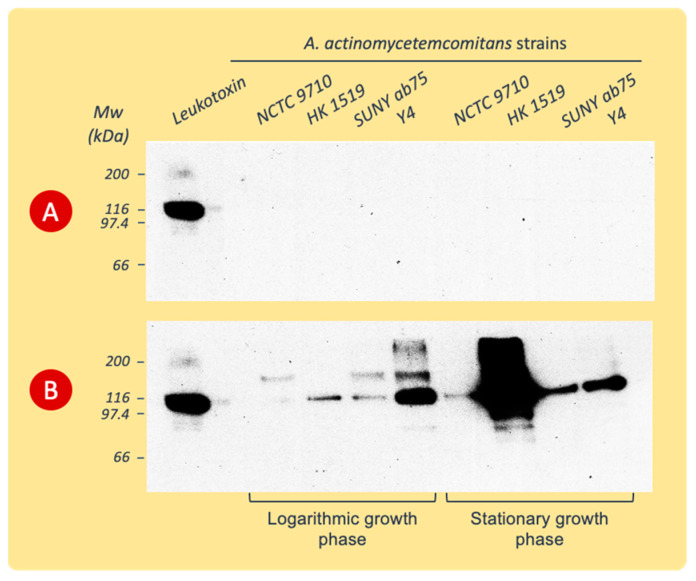
Western blots of proteins precipitated from culture supernatants of various *A. actinomycetemcomitans* strains and separated by SDS-PAGE before being exposed to polyclonal rabbit LtxA antiserum. The supernatants were collected during the logarithmic or stationary phases of growth in PYG (**A**) or BM (**B**) broth. Data from representative experiments are shown.

The immunoblot revealed antiserum reactivity with proteins precipitated only from BM culture supernatants (Figure 4). The antibodies bound to the protein band with the molecular weight of leukotoxin and to protein bands at positions of lower or higher molecular weights ranging between 70 and >200 kDa. 

### 3.4. Screening of Leukotoxicity by the Cytolytic Assay

**Figure 5:**
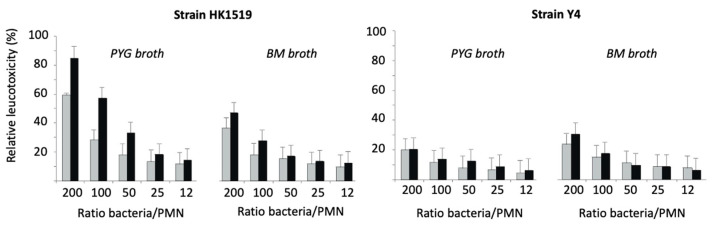
Relative leukotoxicity of *A. actinomycetemcomitans* HK1519 and Y4 grown in PYG or BM broth and harvested during the logarithmic (grey bars) or stationary (black bars) phases of growth. The leukotoxic activity was examined at various ratios of bacteria/PMN.

The LtxA-induced lysis of human PMNs showed that the JP2 genotype (strain HK1519) exhibited the highest activity irrespective of the culture condition (Figure 5). Strain Y4 also showed leukotoxic activity; however, it was lower than that of the JP2 genotype. 

At a ratio of 200 bacteria/PMN, the RLs of HK1519 cells from the logarithmic and stationary growth phases were 60% and 85%, respectively. The corresponding RLs of strain Y4 were lower, ranging from about 20 to 30% (Figure 5).

**Figure 6:**
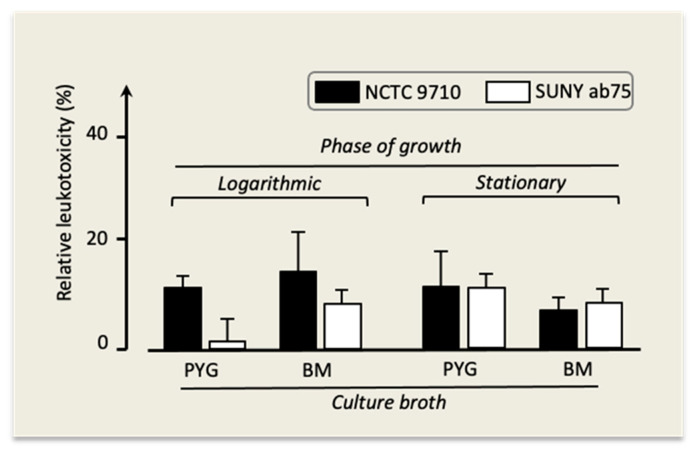
Leukotoxicity of *A. actinomycetemcomitans* strains NCTC 9710 and SUNY ab75 under different growth conditions. Mean ± SD of three replicates.

In all experiments, the leukotoxicity of strains NCTC 9710 and Suny ab75 was somewhat lower compared to the one of strain Y4, and it reached detectable levels under the conditions displayed in Figure 6. 

## 4. Discussion

It is evident from the present findings that the growth conditions may affect the leukotoxicity of *A. actinomycetemcomitans*, which may partly explain the discrepancies in leukotoxin production and leukotoxicity observed for the same strains by various researchers. Both the composition of the culture medium and the phase of growth at which the bacteria were harvested influenced the relative leukotoxicity; however, strain-dependent patterns were found. Although the leukotoxicity of the non-JP2 genotype strains showed a tendency to be slightly increased in cultures from BM broth, the highest leukotoxic activity is to be expected with all strains when grown in the absence of blood or serum proteins, such as in PYG broth, due to the minimal loss of the toxin from the bacterial surface into the culture medium. Except for the JP2 genotype, which continued its high LtxA production during the stationary phase of growth, the other strains exhibited higher or equally high leukotoxic activity during the logarithmic phase than during the stationary phase of growth. It is worth noting that in the studies mentioned above, *A. actinomycetemcomitans* was cultured on agar media supplemented with blood or serum, usually for 48 h, before assaying the leukotoxicity. These conditions probably had a considerable impact on the outcome of these studies. 

The culture broths studied have a chemically non-defined composition and are widely used for the cultivation of oral bacteria. The two broths supported the growth of all strains tested, and no significant difference was found in either the generation time, the duration of the lag phase, or the final cell density when the cultures reached the stationary phase of growth. Thus, the variation in leukotoxicity cannot be attributed to differences in the growth pattern.

The main difference between PYG and BM broth is the presence of hemin, vitamin K, and horse serum in the latter. Judging from the equally good growth of all strains in both media, it may be suggested that these supplements do not cover the nutritional demands of *A. actinomycetemcomitans*, as also shown elsewhere [32]. Serum was thought to support growth and was used in selective media to secure good growth upon primary cultivation; however, the opposite effect was observed [33]. Supplementing BM with these compounds was originally suggested to support the growth of fastidious anaerobes such as *Prevotella* and *Porphyromonas* species, formerly classified as *Bacteroides* species [30,34,35].

Despite the similar growth in the two broths, a consistent difference in the release of LtxA from the bacterial cells appears to occur with all strains, with the release being more extensive in BM cultures. The release of membrane-bound active LtxA, both as free molecules and incorporated in outer membrane vesicles, was previously shown to occur upon a 1 h incubation of the bacteria with human serum or serum albumin at 4 °C, with the release being concentration-dependent [27]. Possibly, the horse serum in BM has a similar effect on the release of the toxin. 

In the previous study [27], the leukotoxic activity of the JP2 genotype was also examined with cultures in PYG supplemented with various concentrations of human serum. Intact LtxA and other protein bands of smaller molecular weight that reacted with LtxA-antiserum were detected both in the samples of culture supernatants and the cell extracts. However, the preparations lacked leukotoxic activity even from cultures with low serum concentrations (5%), which could possibly depend on the degradation and inactivation of LtxA by the components of the human serum. This was not observed in this study, which may indicate important differences between the inactivated horse serum used in BM and the human serum previously studied.

The Y4 strain seems to produce more LtxA, either released in the medium or kept bound to the bacterial cells, when grown in BM than in PYG. As mentioned, a strain-dependent pattern appears to exist in LtxA production, with the pattern being influenced by the medium composition. Possibly, these strains may respond to small molecules and nutritional cues of their environment with an altered virulence expression, as also observed with other periodontopathogens [36]. 

In accordance with previous observations, the JP2 genotype (strain HK1519) of *A. actinomycetemcomitans* exhibited a much higher LtxA production than the non-JP2 genotypes [17]. Moreover, the present results indicate that the enhanced LtxA production by the JP2 genotype is more pronounced during the stationary phase of growth. This strain continued to accumulate LtxA bound to the outer membrane, while the non-JP2 genotype (strain Y4) already reached maximal LtxA content in the late logarithmic growth phase. This is opposite to the large previously reported reduction in LtxA expression in the JP2 genotype when approaching the stationary phase of growth [37]. Differences in the culture conditions may be responsible for the discrepant results, as already mentioned above.

Some authors suggested an enhanced pathogenic potential of *A. actinomycetemcomitans* strains belonging to serotype b due to their increased LtxA production [4,38]. LtxA induces a substantial pro-inflammatory response in macrophages by targeting the NLRP3 inflammasome [7,39]. The genotyping of serotype b isolates revealed a subgroup with high virulence [40]. More recently, a genetic marker, the *cagE* gene, was identified for this subgroup [15], which includes the JP2 genotype and about one-third of the non-JP2 serotype b isolates with shared properties of high LtxA production and pathogenicity [16]. The serotype b strain Y4 lacks the *cagE* gene [16]. This genetic difference between the two serotype b strains HK1519 and Y4 agrees with the large difference in their LtxA production as presently found. It remains to be discovered whether the *cagE* gene has a role in the enhanced *LtxA* production and virulence associated with this genotype [15,16].

Extrapolating these in vitro findings to the clinical situation, it seems plausible to expect that the nutritional environment of the inflamed periodontal pocket favors the production and release of LtxA by *A. actinomycetemcomitans.* Especially, strains characterized as heavy LtxA producers, such as those of the JP2 genotype, might stably express this virulence factor at maximum capacity, irrespective of their growth phase. The high pathogenic potential ascribed to the JP2 genotype [41,42] and to other strains of the *cagE* subgroup is probably potentiated by the enhanced release of the toxin from the bacterial surface, due to the presence of serum compounds. Depending on the proteolytic activity of the environment, the free LtxA may be degraded and lose its toxicity [28,29] or remain intact. The higher the production and release of LtxA, the higher the amount of intact toxin molecules that may diffuse into the neighboring soft tissue and trigger inflammatory reactions or kill defense cells [6,43]. The present study supports this hypothesis of the pathogenic potential of specific *A. actinomycetemcomitans* strains and the possible mechanisms. 

## Figures and Tables

**Table 1:** Composition of the two culture broths.

PYG Medium	BM Medium
Bacto peptone 0.5%	Trypticase (BBL) 1%
Trypticase peptone 0.5%	Proteose peptone (Oxoid) 1%
Glucose 1%	Glucose 0.5%
Yeast extract 1%,	Yeast extract 0.5%
NaCl 1.36 mM	NaCl 1.13 mM
MgSO_4_ 0.03 mM	Sterile horse serum 2%
K_2_HPO_4_ 0.22 mM	Hemin 5 mg/L
KH_2_PO_4_ 0.29 mM	Vitamin K 10 mg/L
CaCl_2_ 0.05 mM	
NaHCO_3_ 4.7 mM	

**Table 2:** Generation time in minutes of various strains of *A. actinomycetemcomitans* in two different culture media. Mean ± standard deviation of 3 experiments with each strain.

*A. actinomycetemcomitans* Strains	PYG Broth	BM Broth
NCTC 9710 (serotype c)	92 ± 9	96 ± 6
HK 1519 (serotype b, JP2 genotype)	108 ± 6	96 ± 27
SUNY ab75 (serotype a)	116 ± 12	94 ± 24
Y4 (serotype b, non-JP2 genotype)	92 ± 9	92 ± 18

**Table 3:** Leukotoxin content of extracts from bacteria cultured in two different media and harvested during the logarithmic or stationary phases of growth. The amount of leukotoxin was determined by densitometric analysis (OD × mm^2^) of the LtxA band in the SDS-PAGE of the extracts. Data from two experiments (Exp. 1 and Exp. 2) with each strain are presented.

Strain	PYG Broth				BM Broth			
	Logarithmic		Stationary		Logarithmic		Stationary	
	Exp. 1	Exp. 2	Exp. 1	Exp. 2	Exp. 1	Exp. 2	Exp. 1	Exp. 2
NCTC 9710	0.14	0.68	0.73	0.67	−0.02	0.94	1.33	0.81
HK1519	2.85	4.72	19.7	8.75	2.54	1.62	7.65	7.10
SUNY ab75	0.25	0.17	0.71	0.68	0.74	0.64	0.91	1.51
Y4	3.81	1.52	1.24	1.61	1.66	1.87	3.08	2.79

## Data Availability

Data are available from the corresponding author, Department of Odontology, Umeå University.
